# The controversy of klotho as a potential biomarker in chronic kidney disease

**DOI:** 10.3389/fphar.2022.931746

**Published:** 2022-09-21

**Authors:** Li-Xia Yu, Sha-Sha Li, Min-Yue Sha, Jia-Wei Kong, Jian-Ming Ye, Qi-Feng Liu

**Affiliations:** ^1^ Department of Nephrology, Affiliated Kunshan Hospital of Jiangsu University, Kunshan, China; ^2^ Clinical Research and Lab Center, Affiliated Kunshan Hospital of Jiangsu University, Kunshan, China

**Keywords:** Klotho, controversy, biomarker, chronic kidney disease, diagnosis, and prognosis

## Abstract

Klotho is an identified longevity gene with beneficial pleiotropic effects on the kidney. Evidence shows that a decline in serum Klotho level occurs in early chronic kidney disease (CKD) and continues as CKD progresses. Klotho deficiency is associated with poor clinical outcomes and CKD mineral bone disorders (CKD-MBD). Klotho has been postulated as a candidate biomarker in the evaluation of CKD. However, the evidence for the clinical significance of the relationship between Klotho and kidney function, CKD stage, adverse kidney and/or non-kidney outcomes, and CKD-MBD remains inconsistent and in some areas, contradictory. Therefore, there is uncertainty as to whether Klotho is a potential biomarker in CKD; a general consensus regarding the clinical significance of Klotho in CKD has not been reached, and there is limited evidence synthesis in this area. To address this, we have systematically assessed the areas of controversy, focusing on the inconsistencies in the evidence base. We used a PICOM strategy to search for relevant studies and the Newcastle–Ottawa Scale scoring to evaluate included publications. We reviewed the inconsistent clinical findings based on the relationship of Klotho with CKD stage, kidney and/or non-kidney adverse outcomes, and CKD-MBD in human studies. Subsequently, we assessed the underlying sources of the controversies and highlighted future directions to resolve these inconsistencies and clarify whether Klotho has a role as a biomarker in clinical practice in CKD.

## Introduction

The Klotho gene was first identified as a novel longevity gene in 1997 (Kuro-o et al., 1997). It exists in three paralogs: αKlotho (referred to as Klotho here), βKlotho, and γKlotho (Ito et al., 2000; Dermaku-Sopjani et al., 2013). Human Klotho protein exists both as membrane Klotho (mKlotho) and soluble Klotho (sKlotho) (Zhong et al., 2020). mKlotho is a single-pass transmembrane protein comprising 1,012 amino acids. The extracellular domain (Kl1 and Kl2) of mKlotho can be shed constitutively by anchored proteases and yields sKlotho (Zhong et al., 2020). sKlotho is also produced by alternative Klotho mRNA splicing (Xu and Sun, 2015). Klotho has pleiotropic renal protective actions, including anti-fibrosis, anti-oxidative stress, anti-inflammation, anti-apoptosis (Hum et al., 2017; Yuan et al., 2022), and modulation of autophagy (Chen and Sun, 2019).

Klotho is strongly expressed in the kidney and the level there is related to its functional state (Wang et al., 2018; Kuro, 2019). Therefore, Klotho deficiency is proposed to be a common feature of kidney diseases (Wang et al., 2018) and has an important role in their pathogenesis and development, including chronic kidney disease (CKD) and related complications. A decreased sKlotho level has been observed in the early stages of CKD, preceding the elevation of serum creatine (Scr) level; the sKlotho level gradually decreases with CKD progression (Shimamura et al., 2012; Liu et al., 2017; Neyra et al., 2020b). More importantly, reduced sKlotho was associated with increased adverse clinical outcomes in CKD patients, including Scr doubling, CKD progression, all-cause mortality, and CKD-mineral bone disorders (CKD-MBD) (Kuro, 2017; Charoenngam et al., 2020; Yang et al., 2020). Therefore, sKlotho is postulated as a promising biomarker in early CKD diagnosis and prognosis.

Nevertheless, there has been a significant controversy regarding whether sKlotho serves as a suitable biomarker in CKD because available clinical evidence on the sKlotho value remains debated and is inconsistent (Akimoto et al., 2012; Seiler et al., 2013; Bob et al., 2019; Valenzuela et al., 2019; Savvoulidis et al., 2020; Desbiens et al., 2022). Indeed, apparent controversies remain regarding the relationship between the sKlotho level and kidney function, CKD stages, adverse clinical outcomes, and CKD-MBD. This, therefore, appears to imply an uncertainty regarding sKlotho as a possible biomarker in CKD and represents an area of continuing investigation. The uncertainty of sKlotho arises from various aspects which need to be summarized and addressed. Given this, this review summarizes available negative clinical studies in these respects and mostly explores possible reasons accounting for these controversies. We aimed to address and resolve this inconsistency, systemically evaluate the clinical significance of sKlotho, and highlight the future research directions prior to applying sKlotho as a potential biomarker in CKD. Here, a PICOM search strategy was employed and the Newcastle–Ottawa Scale (NOS) was used for the quality assessment of included studies in this review (Stang, 2010) (Supplementary File S1). Studies with ≥7 stars were rated as high-quality studies.

## The controversy of sKlotho as a biomarker in clinical nephrology

As a kidney protective protein, sKlotho deficiency is observed to be associated with reduced kidney function, CKD stages, adverse outcomes, and CKD-MBD, indicating a potential role as a biomarker in CKD. However, available clinical studies yielded inconsistent and even contradictory results regarding the correlation of sKlotho with CKD. To some extent, conflicting evidence indicates uncertainty in the application of sKlotho as a biomarker.

### sKlotho level does not consistently correlate with the estimated glomerular filtration

There is increasing evidence that the source of circulatory sKlotho is derived from the kidney (Sakan et al., 2014; Hu et al., 2016; Thongprayoon et al., 2020), indicating the close association between Klotho and kidney diseases. Theoretically, during pathological conditions (damaged kidney, particularly with injured renal tubule), a deficiency (decrease) of this substance occurs. A number of observational studies have demonstrated that systemic sKlotho levels are downregulated in CKD animal models and CKD patients, and it was further reduced as the eGFR declined (Shimamura et al., 2012; Pavik et al., 2013; Sakan et al., 2014; Seo et al., 2015; Liu et al., 2017; Li et al., 2018; Buchanan et al., 2020; Yang et al., 2020). In this context, sKlotho deficiency is considered to be a common characteristic of CKD that is involved in its pathogenesis and development (Neyra et al., 2020a). Subsequently, Klotho is postulated as a potential diagnostic biomarker.

However, there are observational and cross-sectional studies that do not confirm these. Seiler *et al.* investigated the sKlotho level in 321 CKD patients of stages 2–4. The sKlotho level did not differ significantly based on the CKD stage, and the baseline eGFR was not changed significantly according to sKlotho tertiles. No apparent association was found between the eGFR and sKlotho levels by Spearman correlation analysis (Seiler et al., 2013). Akimoto *et al.* also conducted a study to determine whether the sKlotho level was associated with the kidney function. The sKlotho level appeared to be decreased as the kidney function deteriorated in 131 CKD 1- to 5-stage patients. However, the distribution of sKlotho among the CKD stages failed to reach a significant difference, and this association was not found in multiple regression analysis (Akimoto et al., 2012). A recent study performed by Bob *et al.* also demonstrated that the sKlotho level in patients with diabetic kidney disease (DKD) was not correlated with the eGFR (Bob et al., 2019). Interestingly, in this study, patients with an eGFR<60 ml/min had a higher sKlotho level, which was in agreement with the finding of another study that included patients with autosomal dominant polycystic kidney disease (ADPKD) (Sari et al., 2017). Similar inconsistent findings were also shown in other clinical studies (Devaraj et al., 2012; Hage et al., 2014; Scholze et al., 2014; Desbiens et al., 2022). The inconsistent studies were summarized and the average score was 5.5 stars as shown in Table 1. Several studies were not scored because of the invalidity of NOS for cross-sectional studies.

**TABLE 1:** Characteristics of the negative observational studies regarding the relationship of Klotho with kidney function.

First author	Year	Country	N	Age	Samples	eGFR	sKlotho level	Klotho and eGFR	NOS
Desbiens	2022	Canada	159 non-CKD	53 (46–60)	Plasma	90	677 (565–877) ▲	Similar levels of sKlotho between the two groups.	6 stars
			153 CKD	64 (59–67)		55	662 (543–831)		
Bob	2018	Romania	63 DKD	58.13 ± 12	Serum	65.2 ± 32.5	326.36 ± 246.78 ▲	sKlotho level did not correlate with eGFR	6 stars
Scholze	2014	Denmark	24 CKD	68 (59–75)	Serum	31 (21–55)	236 (193–291) ▲	sKlotho concentrations did not differ among CKD stages	
									
Sari	2017	Turkey	76 ADPKD	50.96 ± 15.59	Serum	57.24 ± 33.80	2.92 (0.99–21.97) ★	sKlotho levels were negatively correlated with eGFR	4 stars
			32 controls	49.53 ± 7.32		90.15 ± 20.71	2.04 (0.95–19.98)		
									
Hage	2014	France	60 CKD	46.7 6 ± 16.6	Serum	71.1 ± 29.2	478 (348–658) ▲	sKlotho is not related to kidney function	
									
Devaraj	2012	United States	61 CKD	55 ± 17	Serum	CKD1 or CKD3	67 (43, 119) vs108 (66, 182) ★	sKlotho was increased in CKD and was decreased in diabetics	4 stars
			82 diabetics	37 ± 12		Normal eGFR	81 (45, 141) vs 35 (15, 58) ★		
									
Seiler	2013	Germany	321 CKD	65.5 ± 12.1	Plasma	43.8 ± 15.6	538 (450–666) ▲	sKlotho level not differ across CKD stages.	8 stars
									
Akimoto	2012	Japan	131 CKD	56 ± 18	Serum	46.3 ± 37.5	759.7 (579.5–1,069.1) ▲	Urinary excreted Klotho, not serum Klotho levels associated with eGFR	5 stars

CKD, chronic kidney disease; N, number; DKD, diabetic kidney disease; ▲, pg/ml; ★, ng/ml; ADPKD, autosomal dominant polycystic kidney disease; eGFR, estimated glomerular filtration rate; NOS, Newcastle–Ottawa scale

### sKlotho does not always predict adverse outcomes for pre-dialysis CKD patients

Klotho deficiency is associated with kidney injury and CKD progression. Indeed, a growing number of studies have examined the association of sKlotho with adverse renal or non-renal outcomes, and the majority of these cohort studies demonstrated a close relationship (Kim et al., 2013; Fountoulakis et al., 2018; Liu et al., 2018; Qian et al., 2018; Yang et al., 2020). In this aspect, the sKlotho level is assumed as a prognostic biomarker for adverse clinical outcomes in this population (Liu et al., 2019a).

By contrast, some prospective and retrospective cohort studies showed different results. For example, Seiler et al. (2013) conducted a study to clarify the association of sKlotho with the adverse outcomes in 321 CKD patients followed up for 2.2 ± 0.8 years. Patients were categorized into three groups based on sKlotho tertiles; the composite clinical outcomes were compared among the groups. The clinical outcomes included Scr doubling, renal replacement therapy (RRT), and mortality. The number of patients with event-free survival did not differ among these groups. There was no relationship between the sKlotho level and combined endpoints in either univariate or multivariate Cox regression analyses (hazards ratio [HR] 1.59, 95% confidence interval [CI] 0.12–20.83, *p* = 0.726). This finding suggested that lower sKlotho could not predict combined adverse outcomes (Seiler et al., 2013). Similarly, a study by Qian et al. (2018) reported that the change in the sKlotho level, not the sKlotho level at baseline, was correlated with CKD progression. Similar nonsignificant findings were also demonstrated in other studies (Seiler et al., 2014; Adamska-Tomaszewska et al., 2020). Regarding the relationship of sKlotho with cardiovascular (CV) events or mortality, in the study of Brandenburg et al. (2015), sKlotho was not found to be associated with CV events (HR 1.03, 95%CI, 0.80–1.31, and *p* = 0.845) or all-cause mortality risk (HR 1.14, 95%CI, 0.94–1.38; *p* = 0.187). This study had the largest sample (2,948 participants, 14% of whom had an eGFR<60 ml/min/1.73 m^2^) and the longest term of follow-up (9.9 years). Another recent observational study with a large sample also failed to show any significant association (Ciardullo and Perseghin, 2022). Interestingly, a study performed by Bob et al. (2019) reported that for patients with DKD, a high sKlotho level rather than a low sKlotho level was associated with an annual rapid decline of kidney function, which contradicted the published data. The inconsistent studies were summarized and the average score was 7 stars, as shown in Table 2.

**TABLE 2:** Characteristics of the negative studies regarding the relationship of Klotho with adverse clinical outcomes (low versus high sKlotho level).

Author	Year	Country	Study design	N	Sample	Age	eGFR (ml/min)	Follow-up	Outcomes	Conclusion	NOS
Ciardullo	2022	Italy	Observational	2509 DM	Serum	60.0 ± 0.2	CKD1-5	—	CV events	sKlotho levels were not linked	8 stars
				480 CKD						with CV events	
Adamska	2020	Poland	Prospective	217	Serum	72 ± 11, 72	No data eGFR<60	3 years	Death	sKlotho levels were not related	stars
				80 CKD		50.1 ± 14.0				to long-term outcomes.	
											
Bob	2019	Romania	Retrospective	63 CKD	Serum	58.13 ± 12	65.2 ± 32.5	12 months	△decline of eGFR	Increased sKlotho was linked with rapid annual decline of eGFR	stars
Qian	2018	China	Prospective	112 CKD	Serum	64.5 ± 12.7	37.5 ± 1.9	1.5 years	RRT	Changes in sKlotho level, not baseline sKlotho, correlated with RRT or CV	7 stars
									CV events		
										events	
Brandenburg	2015	Germany	Prospective	2,948	Unknown	63 ± 10	eGFR>90	9.9 years	CV events	Klotho did not predict	7 stars
							eGFR<90		Death	CV events or death	
											
Seiler	2013	Germany	Prospective	312 CKD	Plasma	65.5 ± 12.1	43.8 ± 15.6	2.2 ± 0.8 years	RRT	Lower sKlotho failed to predict	8 stars
									Death	combined adverse outcomes	

N, number; RRT, renal replacement therapy; Scr, serum creatinine; CV, cardiovascular

### sKlotho does not necessarily predict poor prognosis for patients with maintenance hemodialysis

Klotho is a multifunctional factor with various cytoprotective effects (Buchanan et al., 2020). In addition to kidney protective actions, Klotho also exerts beneficial cardiovascular effects, including homeostasis in calcium and phosphate metabolism and suppression of atherosclerosis, vascular calcification (VC), arrhythmia, myocardial fibrosis, and heart failure (Kitagawa et al., 2013; Navarro-Garcia et al., 2020). These disorders have been identified as independent risk factors for CV morbidity and all-cause mortality, particularly in patients receiving maintenance hemodialysis (MHD) (Pichler et al., 2017). Klotho deficiency exacerbates these disease conditions; thus, it is assumed that Klotho deficiency is associated with an elevated risk for morbidity or mortality in MHD patients (Munoz-Castaneda et al., 2020). Clinical studies have been performed to investigate this potential association, and a small number of them revealed an association between a low sKlotho level and more adverse clinical outcomes and have shown it as a prognostic marker for patients on MHD (Cai et al., 2015; Otani-Takei et al., 2015; Marcais et al., 2017; Wei et al., 2019; Yu et al., 2020; Cai et al., 2021).

However, there were a few observational and cohort studies reporting conflicting results. Buiten *et al.* reported that a lower sKlotho level was associated with an increased risk for coronary artery disease and left ventricular dysfunction in 127 dialysis patients; however, this association was lost after adjusting for confounders (Buiten et al., 2014). Moreover, a recent prospective study by Valenzuela et al. (2019) found that a low sKlotho level was correlated with impaired physical performance, but not with all-cause mortality (relative risk 1.6%, 95% CI 0.65–1.35). Similarly, Nowak et al. (2014) found no association between higher sKlotho levels and a lower risk for mortality in 329 MHD patients with sKlotho either as a continuous variable or a categorical variable in multivariable-adjusted analysis after 2.53 years of follow-up (Nowak et al., 2014). Similar inconsistent results were also demonstrated by other recent prospective cohort studies (Zheng et al., 2018; Adamska-Tomaszewska et al., 2020; Erkus et al., 2021). The inconsistent studies were summarized and the average score was 7 stars, as shown in Table 3.

**TABLE 3:** Characteristics of the negative studies regarding the relationship of Klotho with adverse outcomes (Low versus high sKlotho level) in MHD patients.

Author	Year	Country	Study design	N	Sample	Follow-up	Age	Outcomes	Conclusion	NOS
Erkus	2021	Turkey	Observational	136	Serum	—	48.2 ± 17.4	Uremic cardiomyopathy	sKlotho was not associated with uremic	5 stars
							58.9 ± 16.7		cardiomyopathy	
Valenzuela	2019	Spain	Prospective	30	Plasma	18 months	71 ± 9	All-cause mortality	sKlotho levels were not associated	6 stars
									with mortality	
Zheng	2018	China	Prospective	128	Serum	36 months	61.91 ± 15.3	CAC score	sKlotho levels were not associated	8 stars
			Observational					All-cause mortality	with mortality.	
Buiten	2014	United Kingdom	Observational	127	Plasma	—	67 ± 7	AAC + CAC score	sKlotho levels were not associated	8 stars
								LV-dysfunction	with CV events or mortality	
								CAD		
Nowak	2014	Germany	Sectional	239	Plasma	2.53 years	68 ± 14	All-cause mortality	sKlotho levels were not associated	8 stars
			Prospective						with mortality.	

MHD, maintenance hemodialysis; CAC, coronary artery calcification; LV, left ventricular; AF, atrial fibrillation; AAC, abdominal aorta calcification; CAD, coronary artery disease

### sKlotho is not necessarily associated with acute kidney injury to CKD transition

Acute kidney injury (AKI) is a serious syndrome that is associated with an increased risk for morbidity and mortality (Lafrance and Miller, 2010). Mostly, patients with an AKI episode have an elevated risk for CKD development despite AKI recovery (Lafrance and Miller, 2010; Coca et al., 2012). It is of vital importance to screen and validate possible biomarkers to predict, delay, or reverse adverse AKI consequences such as subsequent CKD and end-stage renal disease risks after AKI. sKlotho has powerful renal and extra-renal actions by modulating oxidative stress, inflammation, apoptosis, and fibrogenesis (Hum et al., 2017). Although sKlotho is not filtered by the kidney, it can reach the tubules *via* transcytosis (Hu et al., 2016). This indicates that it directly confers cytoprotective effects on tubular cells and has therapeutic potential in slowing the progression of AKI to CKD. Preclinical data demonstrated that Klotho protein replacement delayed AKI-to-CKD transition by the regulation of autophagy, apoptosis, and endoplasmic reticulum stress (Shi et al., 2016; Liu et al., 2019b). Recently, Neyra et al. (2019) conducted a cohort study to examine the association of sKlotho with AKI outcomes. In total, 45 AKI subjects and 52 controls were enrolled. Per one-fold higher urine sKlotho:creatinine, an 83% reduction in the risk of developing all-cause mortality, RRT, and a >50% decline in the eGFR during a 90-day follow-up was observed. This study was the first to evaluate the predictive value of sKlotho in AKI patients. Again, due to the limited samples and short follow-up, the results should be interpreted with more caution.

These conflicting results indicate that there is uncertainty about whether a decrease in sKlotho level correlates with kidney function and clinical outcomes. Based on the evidence to date, a low sKlotho level may not represent a useful biomarker for CKD diagnosis and prognosis.

### Causes of controversy of sKlotho as a clinical marker in CKD patients

**FIGURE 1:**
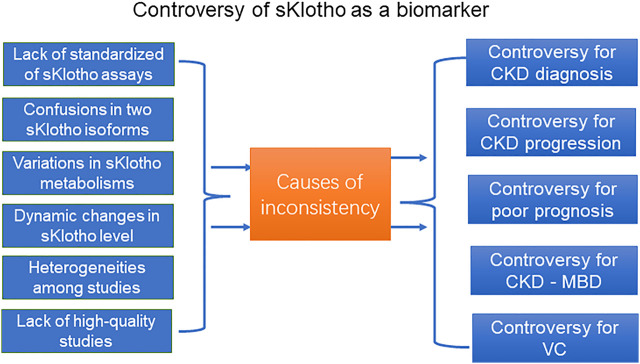
The controversy of sKlotho as a biomarker in CKD.

The present evidence base does not currently support using sKlotho as a biomarker in CKD. Several reasons may contribute to the discrepant results and affect the interpretation of the results (Figure 1).

First, standardization of sKlotho assays has yet to be achieved (Heijboer et al., 2013; Neyra et al., 2020b). The circulating Klotho level can be measured by different immunoassays, including the enzyme-linked immunosorbent assay (ELISA), time-resolved fluorescence immunoassay (TRF), and immunoprecipitation-immunoblot (IP-IB). A previous study evaluated the quality of three frequently utilized ELISA assays. There were substantial heterogeneities with within-run variation ranging from 4% to 32% among three commercial assays, indicating the uncertainty of ELISA assays (Heijboer et al., 2013). As for ELISA and TRF, another report demonstrated sKlotho value using TRF was associated with eGFR and sKlotho value using ELISA was associated with age instead of eGFR, favoring the possible application of TRF assay (Pedersen et al., 2013). Again, for ELISA and IP-IB, a recent study compared the performances of the two assays in determining Klotho measurement. sKlotho level determined by IP-IB displayed a strong correlation with eGFR but minimal correlation with ELISA, suggesting the superior performance of IP-IB assay (Neyra et al., 2020b). However, IP–IB assay also has several weaknesses including more time and effort and restriction to thawed specimens (Neyra et al., 2020b). Despite the better reported performances of the TRF and IP-IB assays, the ELISA assay is usually preferred due to the kit being more rapid and cheaper in clinical practice. Given the significant differences in sensitivity and specificity across available sKlotho assays kits, it is difficult to produce consistent results dependent on the different sKlotho assays kits (Heijboer et al., 2013; Neyra et al., 2020b). The assay-related variance may partly explain the inconsistent results of different authors. On the one hand, sKlotho is cleaved from cell membrane mKlotho by metalloproteinases (ADAM) including ADAM 10 and ADAM17; thereafter, its concentration is influenced by ADAMs. This may be one cause of the increased sKlotho level, while the mKlotho level is decreased in the DKD model (Typiak et al., 2021; Ciardullo and Perseghin, 2022). On the other hand, sKlotho is also generated by alternative mRNA splicing and the existing ELISA assays were unable to distinguish whether sKlotho results from shedding of the extracellular domain of mKlotho or alternative splicing of its transcript. To date, this spliced Klotho transcript has not been determined or its determination is inconclusive based on published data (Jadhav et al., 2021; Li et al., 2021). Additionally, clinical samples for Klotho determination were collected from fasting patients and stored at −80°C after centrifugation until further analysis. The stability of Klotho in different samples and the ideal time point for its analysis affect its quantification. For example, consistency between serum and plasma samples has not been achieved for the same Klotho assay or among different Klotho assays (Heijboer et al., 2013). For urinary sKlotho determination, its concentration in freshly voided urine was significantly higher than that in stored samples, meaning that Klotho is unstable in stored human urine (Adema et al., 2015). Furthermore, the performance of the same assay is limited due to additional freeze–thaw cycles of clinical samples (Neyra et al., 2020b). To produce accurate results, standardization in terms of Klotho assays, sample processing, storage conditions, and time point for analysis should be developed.

Second, it remains unclear what happens regarding Klotho production and clearance in the failing kidney. This may be another reason for the inconsistent findings in specific CKD models such as DKD versus non-DKD (Wang et al., 2018; Bob et al., 2019), pre-dialysis versus dialysis (Liu et al., 2019a; Valenzuela et al., 2019), remaining kidney function versus urine output (Golembiewska et al., 2013). In addition to non-renal Klotho production, the tubule may play an important role in maintaining the sKlotho level. The fact that sKlotho was detected in urine indicates that renal epithelial cells were involved in Klotho metabolism because Klotho is too large to pass through the glomerular barrier (Hu et al., 2010). It has been reported that sKlotho undergoes a transcytosis process and reaches the tubular lumen (Hu et al., 2016). This may explain the fact that serum sKlotho was inversely associated with urine output other than with the remaining kidney function in patients undergoing peritoneal dialysis (Sikorska et al., 2019). Tubular injury decreased sKlotho clearance and was associated with an increased serum sKlotho level in specific CKD models, such as DKD (Bob et al., 2019). This indicates serum sKlotho may not serve as a candidate marker for eGFR, but instead, as a possible marker for renal tubular damage, which may be another possible reason for the conflicting results.

Third, the sKlotho level is regulated by several pathological processes, including inflammation, oxidative stress, uremic toxins, and the renin-angiotensin system (RAS), as well as commonly administrated agents, such as phosphate binders or active vitamin D in CKD (Kale et al., 2021; Xia and Cao, 2021). These pathological states and used drugs are common in CKD, but also different according to the CKD stage, which may confound their relationship. Additionally, the sKlotho level was influenced by dialysis modalities in dialysis patients (Hu et al., 2016; Picciotto et al., 2019). Therefore, the sKlotho level varies over time. A single measurement of the sKlotho level at baseline does not reflect the actual sKlotho level.

Fourth, sKlotho is also produced by extra-renal organs (Lim et al., 2015; Picciotto et al., 2019). Extra-renal Klotho production is probably stimulated as a compensatory source under CKD conditions (Kale et al., 2021). The contribution of extra-renal organs such as the parathyroid glands, spleen, and choroid plexus to systemic sKlotho remains a matter of debate (Picciotto et al., 2019). This indicated that the association between the sKlotho level and kidney function may be underestimated due to extra-renal organ sKlotho production.

Finally, the quality of the included studies inevitably affects the interpretation of the results. Regarding the correlation of sKlotho with the eGFR, the design of the cited literature is observational or case-control (Table 1). These study types have inherent limitations, and the study quality is relatively low (5.5 stars). Concerning the sKlotho level in adverse outcomes, the design of the literature is a prospective or retrospective cohort, and the study quality is relatively high (7 stars) (Tables 2, 3). Despite this, there are still significant differences, particularly in terms of specific CKD etiology, sample size, follow-up, sample types, and endpoints among the cohort studies (Tables 2–4). Given this, it may not be reasonable to combine these studies to produce more convincing results, or one study may be more rigorous than another study under specific conditions.

**TABLE 4:** Characteristics of the negative studies regarding the relationship of Klotho with vascular calcification in CKD patients.

Author	Year	Country	Study design	N	Sample	Age	Disease models	Outcomes	Relationship	NOS
Liang	2021	China	Observational	716	Serum	53.6 ± 13.5 (men)	General population	BP cfPWV	No	7 stars
						51.0 ± 12.0 (women)				
Savvoulidis	2020	Greece	Observational	60	Serum	63 (52, 72.5)	CKD1-5	CAC	No	7 stars
								AVC		
Chou	2019	Taiwan	Before and after	62	Serum	59 (52–65)	MHD	AAC	No	—
Nattero Chávez	2019	Spain	Observational	164	Plasma	37 ± 10	DM	MAC	No	6 stars
Krishnasamy	2017	Australia	Prospective	82	Serum	62.9 ± 10.2	CKD4-5	AAC	No	8 stars
							42Controls			
Di Lullo	2015	Italy	Observational	100	Serum	51 (46–56)	CKD3-4	Valve Calcification	No	5 stars
Morita	2015	Japan	Observational	157	Serum	W:65.8 ± 11.5	CKD2	CAC	No	7 stars
						M:67.0 ± 11.6	CKD2	AVC	No	
Buiten	2014	United Kingdom	Observational	127	Plasma	67 ± 7	MHD	AAC	No	8 stars
								CAC	No	
Kitagawa	2013	Japan	Observational	114	Serum	58 (47–66)	CKD1-3	ACI	No	5 stars

CKD, chronic kidney disease; BP, blood pressure; cfPWV, carotid–femoral pulse wave velocity; MHD, maintenance hemodialysis; W, women; M, men; MAC, medial arterial calcification; CAC, coronary artery calcification; AVC, aortic valve calcification; AAC, abdominal aorta calcification; ACI, aortic calcification index

Taken together, there are conflicting results from various sources, such as the uncertainty of sKlotho assays or Klotho production/clearance and differences in disease conditions, treatment modalities, drugs, and study quality. Consequently, these inevitably challenge the translation of sKlotho into clinical practice. Therefore, the current evidence should be interpreted with caution until further studies are reported.

### sKlotho was not definitely associated with vascular calcification

Vascular calcification (VC), which results from excess calcium phosphate deposition in blood vessels and/or heart valves, is a hallmark feature of CKD-MBD (Hu et al., 2011). It is highly prevalent in several diseases such as CKD and diabetes mellitus, and in aging (Yannoutsos et al., 2018; Timofte et al., 2020). It has been demonstrated that VC contributes to an increased risk for CV morbidity and all-cause mortality and was identified both as an independent predictor of poor clinical outcomes and an interventional target for the CKD population (Rennenberg et al., 2009; Lioufas et al., 2020).

Klotho systemic deficiency is associated with severe VC. Experimental studies have shown that increased Klotho expression or Klotho therapy ameliorates VC (Hu et al., 2011; Lin and Sun, 2022), indicating that Klotho is implicated in the pathogenesis and progression of VC (Yamada and Giachelli, 2017). Klotho is expressed in vascular cells, and CKD is associated with a deficiency of vascular Klotho (Lim et al., 2012). Local Klotho deficiency in the vasculature can potentiate VC (Lim et al., 2012). Mechanistically, Klotho regulates the osteogenic transition of vascular smooth muscle cells and ameliorates VC by inhibiting phosphate transporter (Pit)-1 and Pit-2 activity (Hu et al., 2011) or by suppressing the Wnt/β-catenin signaling pathway (Chen et al., 2015). Indeed, in the clinic, reduced sKlotho levels have been associated with greater VC, including coronary artery calcification (CAC) (Zheng et al., 2018; Koga et al., 2020), aortic valve calcification (AVC) (Chen et al., 2021), and abdominal aortic calcification (AAC) (Cai et al., 2015; Savvoulidis et al., 2020; Orces, 2022). A reduced sKlotho level was also correlated with vascular dysfunction, including arterial stiffness in CKD patients in multivariate analyses (Kitagawa et al., 2013; Memmos et al., 2019). These data suggested that sKlotho has a beneficial role against VC and is thus presumed as a surrogate biomarker for VC.

By contrast, some observational and cross-sectional studies reported no association between sKlotho and AVC, CAC, or AAC in CKD patients, indicating the controversy regarding the role of sKlotho in mediating VC. A previous study which enrolled 127 patients with MHD reported that the serum sKlotho level was not related to the AAC or CAC scores neither in a crude model nor adjusted model (Buiten et al., 2014). Another study reported that patients with CKD had a higher prevalence of AAC compared with controls without CKD, yet no significant changes in the sKlotho levels between the two groups were observed (Krishnasamy et al., 2017). Additionally, the clinical irrelevance of sKlotho in AAC was demonstrated in this study in a multivariate regression analysis (Krishnasamy et al., 2017). A recent study conducted by Savvoulidis et al. (2020) found that there was no association of sKlotho with CAC or AVC by multivariate analysis, although the sKlotho level in patients with stage-5 CKD was dramatically reduced in comparison with that in patients with stage-3 CKD (Savvoulidis et al., 2020). Similar negative results were also verified in other studies (Di Lullo et al., 2015; Meuwese et al., 2015; Baralić et al., 2019). Interestingly, two other studies which enrolled CKD patients with moderately impaired kidney function reported that the sKlotho levels were positively correlated with CAC in coronary artery biopsies (van Venrooij et al., 2014) or AVC in men after adjustment for confounders in the subgroup analysis despite no association in the overall analysis (Morita et al., 2015). For subjects with normal kidney function, no significant relationship between sKlotho and arterial stiffness or VC was also observed (Nattero-Chávez et al., 2019; Liang et al., 2021). Furthermore, an interventional clinical trial revealed recently the amelioration of AAC was not followed by an increase or decrease in the sKlotho level in MHD patients with secondary hyperparathyroidism, which further challenged the clinical value of sKlotho in VC (Chou et al., 2019) The inconsistent studies were summarized and the average score was 6.6 stars, as shown in Table 4. Given this, the above inconsistent evidence means that the association between sKlotho and VC remains to be determined.

These discrepant findings represented a more complex role of Klotho in VC. There were several explanations for the conflicting results. First, whether Klotho is located in the vasculature is a matter of debate (Mencke et al., 2015). Although the differences in antibodies against Klotho isoforms may partially account for the discrepant findings (Lewin and Olgaard, 2015), the effect of systemic Klotho on local vasculature is uncertain. Second, the association of sKlotho with VC may differ depending on specific disease conditions, and the differences in participant baseline characteristics may complicate the results. For example, the prevalence of VC in CKD increases as CKD progresses, and it is higher in dialysis patients who have a greater risk for VC than those for pre-dialysis (Cai et al., 2015; Krishnasamy et al., 2017). Additionally, locations and diagnostic strategies for VC confuse the interpretations. Furthermore, the precise molecular mechanism underlying VC is complex and not fully elucidated, and the roles of other regulators in this process should not be precluded (Kurabayashi, 2019). Most importantly, available data examining their association arise from cross-sectional or observational studies, not retrospective or prospective cohort studies. These studies are unable to supply strong evidence to clarify whether their causal link is due to inherent defects, such as selection bias, and other potential confounders (6.6 stars). Therefore, it is not possible to conclude whether or not reduced sKlotho is associated with greater VC due to the conflicting results or a lack of prospective cohort studies, although this notion was confirmed by pre-clinical studies. The significance of sKlotho in suppressing VC formation needs to be re-evaluated in prospective cohort studies in the future.

Consequently, due to the above contradictions and insufficient data, the association of sKlotho with VC remains uncertain and needs to be clarified in the future.

### sKlotho may not be an ideal biomarker for CKD-mineral bone disorders

**FIGURE 2:**
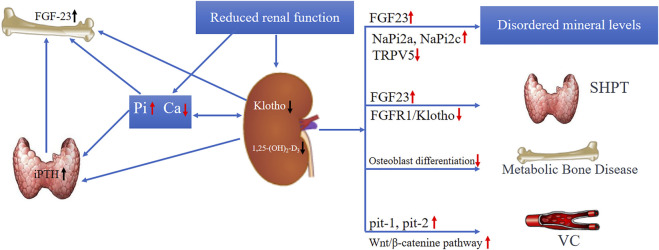
The role of Klotho in CKD-MBD.

Serum calcium and phosphate levels are regulated by 1,25-dihydroxyviatmin D3, intact parathyroid hormone (iPTH), and calcitonin by balancing their intestinal uptake, renal excretion, and bone mobilization. Klotho-deficient mice exhibited mineral metabolism disorders, including phosphate retention, hypercalcemia; VC, valve calcification; and elevated fibroblastic growth factor-23 (FGF23) levels (Kuro-o et al., 1997). These disorders are ubiquitous in CKD; thus, diminished Klotho is supposed to be implicated in the modulation of CKD-MBD (Figure 2) (Neyra et al., 2020a). Klotho downregulation antedated FGF23, iPTH, and phosphate elevation in human CKD (Shimamura et al., 2012; Rotondi et al., 2015; Khodeir et al., 2019). This means that Klotho may be a possible early biomarker of CKD-MBD (Kuro, 2017). Indeed, Klotho modulates calcium and phosphate metabolism primarily *via* FGF23-dependent mechanisms as well as non-FGF23-dependent mechanisms (Kawai, 2016; Andrukhova et al., 2017). The net effect of Klotho on mineral metabolism is to maintain the serum calcium level, but to decrease the serum phosphate level (Figure 2) (Neyra et al., 2020a). In agreement with this, Klotho was positively related with calcium and inversely related with phosphate in many clinical studies (Kim et al., 2013; Kitagawa et al., 2013; Sakan et al., 2014; Rotondi et al., 2015; Sawires et al., 2015; Liu et al., 2017; Savvoulidis et al., 2020).

Despite this, there is evidence available that does not support this view. Regarding calcium metabolism, Klotho was reported to increase its reabsorption and reverse renal calcium loss by modulating renal calcium-selective channels independent of FGF23 (Wolf et al., 2014; Wright et al., 2019). This effect of Klotho results in normal or near-normal calcium levels. However, Klotho was also reported to reduce calcium absorption and contribute to a decreased calcium level by inhibiting the production of active vitamin D and iPTH *via* FGF23 (Yoshida et al., 2002; Hu et al., 2013; Olauson et al., 2013). With respect to phosphate metabolism, Klotho is believed to lower hyperphosphatemia by stimulating renal phosphate excretion and maintaining phosphate homeostasis. A recent study reported that sKlotho may be a new biomarker of phosphate reabsorption after adjustment for confounders, including FGF23. Yet, in this study, Klotho was observed to be positively associated with phosphate reabsorption (Tan et al., 2017), and this means that Klotho inhibited phosphate excretion, which was contrary to the published data. Other studies also demonstrated no negative relationship between Klotho and the phosphate level (Morita et al., 2015; Hage et al., 2016). In the case of iPTH, Klotho and FGF receptor 1 are co-located in the parathyroid, which suggests that the parathyroid is a Klotho-targeted organ (Yan et al., 2015). Generally, Klotho mediated the suppression of iPTH by several pathways, including canonical Klotho-FGF23 signaling (Koizumi et al., 2013; Olauson et al., 2013; Fan et al., 2018). Nevertheless, Klotho was also reported to induce iPTH secretion by regulating parathyroid Na^+^ and K^+^-ATPase activity (Imura et al., 2007). Klotho may also have an indirect role in triggering PTH secretion by directly regulating mineral metabolism and 1,25 (OH) 2D synthesis (Kimura et al., 2016). Moreover, this paradoxical relationship of Klotho with iPTH was found in clinical studies (Buiten et al., 2014; Dhayat et al., 2020). Regarding the effect of Klotho on bone metabolism, it was found to be expressed in osteocytes (Rhee et al., 2011), and its expression was downregulated in renal osteodystrophy in CKD (Komaba et al., 2017). Klotho-deficient mice developed osteoporosis or retardation in bone resorption (Kawaguchi et al., 1999; Yamashita et al., 2000), while sKlotho delivery may induce bone differentiation and promote bone mineralization in osteoblast cells (Toan et al., 2020). Surprisingly, unlike systemic Klotho deletion, its specific depletion from osteocytes may dramatically increase bone formation and Klotho overexpression in osteoblastic cell-suppressed bone mineral formation and osteogenic activity (Komaba et al., 2017). The complicated role of Klotho on bone metabolism possibly leads to inconsistent results regarding the association of Klotho with bone fracture in clinical studies (Chalhoub et al., 2016; Ribeiro et al., 2020; Desbiens et al., 2022).

The inconclusive results represent an important controversy in this area. These discrepancies may result from different experimental animals, the CKD stage, diet composition, and varied participant baseline characteristics (Yokoyama et al., 2012; Morita et al., 2015; Zhao et al., 2015; Dhayat et al., 2020). Additionally, it is difficult to specifically clarify the interplay of Klotho with other regulators such as FGF23, vitamin D, and iPTH in CKD-MBD, and this is possibly responsible for the confusion. To elucidate the more complicated role of sKlotho in the regulation of CKD-MBD, further investigation is necessary.

## How to resolve the controversy

sKlotho is postulated to become a potential marker in CKD; however, one should be aware that there are still contradictory findings in terms of the relationship of sKlotho with kidney function, CKD stage or progression, and CKD-MBD. This indicates that a complete consensus has not been reached in this context due to the existing inconsistent results. To address this issue, the sources of controversies previously described should be resolved specifically before the potential application of sKlotho as a biomarker in CKD.

First, one important issue is how to accurately detect sKlotho in serum or body fluid. Several available sKlotho assays failed to precisely determine the actual sKlotho level or the range of the sKlotho reference value (Heijboer et al., 2013; Neyra et al., 2020b). Interestingly, a recent study by Espuch-Oliver et al. (2022) investigated the reference values of serum sKlotho in a larger sample with 346 healthy adults by ELISA. They observed that sKlotho levels differed significantly across ages, and sKlotho was inversely correlated with age in healthy subjects. This study provided the possibility for the determination of the sKlotho reference value in the future. Better or standardized methods for Klotho measurement are anticipated to yield more reliable results. In agreement with this, a novel sKlotho assay (IP-IB) was shown to display better performance than other available sKlotho assays, which may resolve this issue in the future (Neyra et al., 2020b). One other important question regarding detecting the sKlotho level is how to distinguish the sources of sKlotho due to the presence of two isoforms in the circulation. It has been verified that sKlotho is generated mainly from mKlotho and shed extracellularly by ADAM10 and ADAM17 action. Therefore, the expressions and activities of ADAMs in different CKD models may influence sKlotho production (Akasaka-Manya et al., 2020). Consequently, in addition to the sKlotho level, ADAM10 and ADAM17 should also be determined and compared in the specific CKD context. In addition, the secreted sKlotho isoform that is produced by alternative mRNA splicing comprises a unique 15aa sequence at the C-terminus in the Kl1 domain; given this, a novel antibody has recently been generated to specifically detect this secreted sKlotho isoform (Jadhav et al., 2021). Consequently, this recently reported novel assay offers a possibility for resolving this issue in the future. Another important issue is that the sKlotho level changes over time due to the presence of a cluster of regulators in CKD, including vitamin D, phosphate binders, inflammation, oxidative stress, and RAS. Furthermore, part of the total sKlotho detected in serum is also derived from other non-kidney organs, and this may alter the association of Klotho with kidney function. Therefore, to achieve a more accurate result, sKlotho measurement should be conducted repeatedly and averaged, and the sKlotho value should be obtained after eliminating or balancing the influence of other potential regulators, including inflammation, drugs, RAS and extra-kidney organs, in the study design.

Second, the kinetics of sKlotho is currently still not fully understood (Hu et al., 2016; Zhong et al., 2020). It was reported that ^125^I-labeled exogenous Klotho is located mainly in the kidney, with much lower levels in other organs, including the spleen, liver, heart, and brain (Hu et al., 2016). The half-life of exogenous Klotho was much longer in anephric rats compared with normal rats. Moreover, Klotho was also detected in urine because it can enter the urinary lumen *via* transcytosis by renal proximal tubules (Hu et al., 2016). Therefore, a healthy kidney plays a vital role in maintaining the Klotho balance (Lim et al., 2015; Hu et al., 2016). Not surprisingly, sKlotho production, distribution, and clearance varied significantly under normal and CKD conditions, particularly the condition with complete kidney loss (Hu et al., 2016; Picciotto et al., 2019). In addition, sKlotho production/clearance may be significantly different even in a specific CKD model. For example, Picciotto *et al.* recently reported that for CKD patients with an eGFR<60 ml/min, Klotho was also cleared by splanchnic organs (Picciotto et al., 2019). This indicated that the association of sKlotho with CKD may differ depending on the particular CKD model (Akimoto et al., 2012; Sari et al., 2017; Bob et al., 2019), as well as in dialysis versus pre-dialysis (Golembiewska et al., 2013; Liu et al., 2018). Therefore, the kinetics of sKlotho both in a specific CKD model and at different CKD stages are required to be intensively investigated and characterized in the future. On the basis of fully understanding the metabolic kinetics of Klotho, this association should be examined carefully under the condition of a specific CKD.

Finally, due to the current controversy, high-quality studies, such as interventional trials and prospective or retrospective cohort studies, with standard diagnostic criteria and indicators, are warranted to elaborate and clarify the confusion of Klotho in CKD. In this regard, meta-analysis and systemic review also offer the potential possibility of addressing this issue. Indeed, several meta-analyses have been performed to address this issue in recent years. These published studies supported a close association of sKlotho with kidney function and adverse outcomes in CKD (Wang et al., 2018; Liu et al., 2019a; Liu et al., 2020; Liu et al., 2021). However, the meta-analyses included a very limited number of eligible studies, with small samples. Above all, there were substantial heterogeneities among the included studies, indicating the differences in terms of participant baselines, study designs, and statistical methods, and thus, the conclusion drawn may to some extent not be convincing. Additionally, it must be noted that an individual biomarker, for example, sKlotho, inherently has some limitations for accurate diagnosis and monitoring progression in CKD without the combination of a set of other biomarkers because a single indicator lacks sufficient performance and efficiency to reflect the complexity of the mechanisms underlying CKD pathogenesis and development. Due to the limitations, clinical predictive models incorporating Klotho and other traditional or novel indicators possibly have the greatest potential to evaluate the clinical utility of Klotho (Manou et al., 2020; Yan et al., 2021). Therefore, to gain more information regarding this association, additional high-quality studies in these respects should be conducted, and only in this way can current disputes be addressed and specifically resolved in the future.

## Conclusion and perspectives

CKD is increasingly identified as a global threat to public health, and screening and validating surrogate biomarkers in CKD is critical for its management. Klotho is involved in various biological processes. Its role in clinical nephrology has been particularly examined over recent years. Despite a significant association between sKlotho and kidney function, for CKD outcomes and CKD-MBD, as demonstrated by the experimental and epidemiological studies, there are equally inconsistent findings that suggested that sKlotho may not be a good surrogate biomarker in CKD. This means that much still needs to be resolved regarding the clinical significance of Klotho in CKD. This prompts us to continuously seek the possible sources of controversy and specifically address and resolve this issue in the future. Given the conflicting results, further well-designed research is urgently required to clarify and validate the clinical value of Klotho. In addition, more efforts should be directed to improving sKlotho assay performance. Through this, the possibility of Klotho as a potential biomarker is to be systemically assessed, and a more reliable conclusion can be reached.
