# Supplementary material for: The controversy of klotho as a potential biomarker in chronic kidney disease
Source: Front Pharmacol. 2022 Sep 21;13:931746. doi: 10.3389/fphar.2022.931746 (PMC9532967; doi:10.3389/fphar.2022.931746)
Supplement: Supplementary file 1 [file DataSheet1.docx]

**Methods**

We employed a PICOM search strategy in PubMed database as follows:

**1.sKlotho and estimated** **Glomerular Filtration（eGFR）**

**Patients:** chronic kidney disease or chronic nephropathy or chronic kidney insufficiency or CKD or pre-dialysis

**Intervention:** Klotho or α-Klotho or sKlotho

**Comparison:** Klotho level across CKD stages

**Outcomes:** association of Klotho level and kidney function or eGFR

**Methods**: cross-sectional or case-control study or observational study

**2.sKlotho and adverse outcomes in pre-dialysis**

**Patients:** chronic kidney disease or chronic nephropathy or chronic kidney insufficiency or CKD or pre-dialysis

**Intervention:** Klotho or α-Klotho or sKlotho

**Comparison:** high Klotho level vs. low Klotho level

**Outcomes:** morbidity or mortality or prognosis or outcome or kidney function or eGFR decline or kidney function deterioration or CKD progression

**Methods**: retrospective or prospective cohort studies or observational study

**3.sKlotho and adverse outcomes in** **hemodialysis**

**Patients:** chronic kidney disease or end stage renal disease or end stage kidney disease or dialysis or uremia or hemodialysis or renal dialysis

**Intervention:** Klotho or α-Klotho or sKlotho

**Comparison:** high Klotho level vs. low Klotho level

**Outcomes:** morbidity or mortality or cardiovascular event or cardiovascular disease or coronary artery disease or prognosis or outcome or survival

**Methods**: retrospective or prospective cohort studies or observational study

**4.sKlotho and vascular calcification**

**Patients:** chronic kidney disease or chronic nephropathy or chronic kidney insufficiency or CKD or end stage renal disease or end stage kidney disease or uremic or uremia or dialysis or renal dialysis

**Intervention:** Klotho or α-Klotho or sKlotho

**Comparison:** high Klotho level vs. low Klotho level

**Outcomes:** calcification or vascular calcification or blood vessel calcification or arterial calcification or artery calcification or valve calcification or heart calcification

**Methods**: cross-sectional study or case-control study or observational study or cohort study
